# The *Angiostrongylus vasorum* Excretory/Secretory and Surface Proteome Contains Putative Modulators of the Host Coagulation

**DOI:** 10.3389/fcimb.2021.753320

**Published:** 2021-11-02

**Authors:** Nina Germitsch, Tobias Kockmann, Lars M. Asmis, Lucienne Tritten, Manuela Schnyder

**Affiliations:** ^1^ Institute of Parasitology, Vetsuisse Faculty, University of Zurich, Zurich, Switzerland; ^2^ Graduate School for Cellular and Biomedical Sciences, University of Bern, Bern, Switzerland; ^3^ Functional Genomics Center Zurich, Swiss Federal Institute of Technology Zurich (ETH Zurich), University of Zurich, Zurich, Switzerland; ^4^ Center for Perioperative Thrombosis and Hemostasis, Zurich, Switzerland

**Keywords:** *Angiostrongylus vasorum*, proteomics, excretory/secretory proteins, cuticular surface, coagulation, fibrinolysis, ROTEM, endothelial cells

## Abstract

*Angiostrongylus vasorum* is a cardiopulmonary nematode of canids and is, among others, associated with bleeding disorders in dogs. The pathogenesis of such coagulopathies remains unclear. A deep proteomic characterization of sex specific *A. vasorum* excretory/secretory proteins (ESP) and of cuticular surface proteins was performed, and the effect of ESP on host coagulation and fibrinolysis was evaluated *in vitro*. Proteins were quantified by liquid chromatography coupled to mass spectrometry and functionally characterized through gene ontology and pathway enrichment analysis. In total, 1069 ESP (944 from female and 959 from male specimens) and 1195 surface proteins (705 and 1135, respectively) were identified. Among these were putative modulators of host coagulation, e.g., von Willebrand factor type D domain protein orthologues as well as several proteases, including serine type proteases, protease inhibitors and proteasome subunits. The effect of ESP on dog coagulation and fibrinolysis was evaluated on canine endothelial cells and by rotational thromboelastometry (ROTEM). After stimulation with ESP, tissue factor and serpin E1 transcript expression increased. ROTEM revealed minimal interaction of ESP with dog blood and ESP did not influence the onset of fibrinolysis, leading to the conclusion that *Angiostrongylus vasorum* ESP and surface proteins are not solely responsible for bleeding in dogs and that the interaction with the host’s vascular hemostasis is limited. It is likely that coagulopathies in *A. vasorum* infected dogs are the result of a multifactorial response of the host to this parasitic infection.

## 1 Introduction

*Angiostrongylus vasorum* is a cardiopulmonary nematode of canids and is focally endemic in Europe, South America and Canada (Koch and Willesen, 2009; Jefferies et al., 2010; Priest et al., 2018). In many European countries *A. vasorum* has been increasingly reported in the last two decades and is considered an emerging parasite (Maksimov et al., 2017; Gillis-Germitsch et al., 2020; Fuehrer et al., 2021). Adult stages reside in the right side of the heart and the pulmonary artery of the definitive host (Guilhon and Cens, 1973) where they are in contact with the endothelium. Definitive hosts get infected by ingesting intermediate hosts (slugs and snails) harboring infectious third stage larvae (L3) but may also acquire the infection through ingestion of paratenic hosts or through direct uptake of L3 released from gastropods (Guilhon and Cens, 1973; Robbins et al., 2021).

The most common clinical signs upon canine angiostrongylosis are respiratory issues, such as increased respiration rate, coughing and dyspnea, caused by migrating larvae, which induce inflammatory reactions and damage to lung tissue (Koch and Willesen, 2009; Schnyder et al., 2010). In addition, approximately one third of infected individuals show internal or open bleeding (Adamantos et al., 2015; Glaus et al., 2016), and few develop neurological disorders, usually caused by cerebral or spinal bleeding (Wessmann et al., 2006). Once bleeding develops, the infection is often fatal (Chapman et al., 2004; Adamantos et al., 2015; Sigrist et al., 2017; De Zan et al., 2021; Tachmazidou et al., 2021). Bleeding dogs are usually hypocoagulable and show hyperfibrinolysis (Adamantos et al., 2015; Sigrist et al., 2017). Bleeding has been associated with alteration of various coagulation parameters and was attributed to thrombocytopenia, increased or decreased anti- or procoagulant factor activity, or disseminated intravascular coagulation (DIC) (Schelling et al., 1986; Ramsey et al., 1996; Adamantos et al., 2015; Glaus et al., 2016; Sigrist et al., 2017). A recent study conducted on sera of experimentally infected dogs showed that the complement and coagulation pathways were significantly affected over the course of infection, with reduced levels of relevant coagulation components (Tritten et al., 2021).

Parasites are masters in escaping host immunity and surviving for years within the host. Their long lifespan is accounted for their efficient immune evasion strategies and the creation of an anti-inflammatory environment (Maizels et al., 2004). This is necessary because it protects them from elimination and minimizes severe pathology in the host; nevertheless complications due to immunopathological response can occur (Maizels and Yazdanbakhsh, 2003). Excretory/secretory proteins (ESP) are the best characterized mediators of host immunomodulation by helminths to date. They are released from live parasites from the gut, reproductive organs, the cuticular or tegumental surface, or *via* specialized structures (Rhoads, 1981; Maizels et al., 1982; Lightowlers and Rickard, 1988). ESP contribute to immune evasion by e.g., modulating complement and inflammatory responses, hence allowing the parasite to avoid effects of the host immune response (Lightowlers and Rickard, 1988). Among nematodes, the active and continuous shedding of surface proteins is one source of ESP. ESP have been characterized from a variety of helminths, including from their different developmental stages (Hewitson et al., 2008; Liu et al., 2009; Geary et al., 2012; Sotillo et al., 2014; Morante et al., 2017; Di Maggio et al., 2019). Blood dwelling parasitic helminths and their interference with the mammalian hosts are of particular interest (Simón et al., 2007; Mebius et al., 2013): they disrupt blood flow, while interfering with the host’s coagulation in the right balance to neither induce or suppress blood clotting nor bleeding.

The conclusive mechanisms and pathogenesis behind bleeding disorders induced by *A. vasorum* infections are still poorly understood and under debate. This study reports the results of a deep characterization of *A. vasorum* proteins expressed at the host-parasite interface, namely ESP and cuticular surface proteins, and investigates how ESP may interact with the host and lead to the onset of coagulation disorders.

## 2 Material and Methods

### 2.1 Characterization of *A. vasorum* Excretory/Secretory Proteins and Cuticular Surface Proteins

#### 2.1.1 Parasite Collection

**Figure 1:**
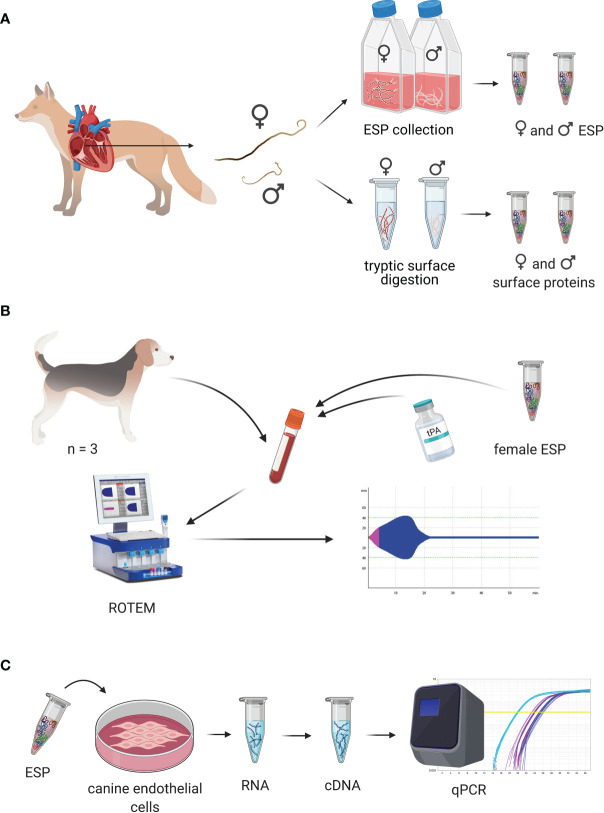
Experimental design of the study. **(A)** Experimental design for collection of excretory/secretory proteins (ESP) and cuticular surface proteins. Collection of *Angiostrongylus vasorum* specimens from hearts of hunted foxes. Females and males were taken into culture separately for collection of female and male ESP. Cuticular surface proteins from female and male specimens were obtained by digestion of their surface. **(B)** Experimental design of coagulation trials. Blood was collected from healthy dogs and *A. vasorum* ESP and different concentrations of tissue plasminogen activator (tPA) were added to dog blood. Coagulation and fibrinolysis in the spiked blood samples was measured using rotational thromboelastometry (ROTEM). **(C)** Experimental design for cell stimulation trials. *A*. *vasorum* ESP was used to stimulate canine endothelial cells. RNA was collected, reversely transcribed and transcript expression of coagulation and fibrinolysis factors measured by qPCR. This figure was designed using BioRender.com.

Live adult *A. vasorum* specimens were collected from lungs and hearts of 93 freshly hunted foxes from the Canton of Zurich, Switzerland (Gillis-Germitsch et al., 2020). The right side of the heart and the pulmonary arteries were opened with sterile surgical scissors. A few drops of blood from the heart were collected in a small sterile Petri dish. Parasite specimens were transferred into the Petri dish and incubated at 37°C to revitalize the parasites. Parasites were then washed 3 times in sterile pre-warmed phosphate-buffered saline (PBS) and identified as *A. vasorum* according to their size and morphology (reproductive organs wrapped around red intestine in females and bursa copulatrix with two spicules and spinal tail curve in males) (Guilhon and Cens, 1973). Females and males were processed separately, easily distinguishable due to their sexual dimorphism (**Figure 1A**). Motile worms were transferred to RPMI 1640 medium (Gibco, Thermo Fisher Scientific) containing antibiotic-antimycotic solution (500 units penicillin, 0.5 mg streptomycin and 1.25 μg amphotericin B per ml, Thermo Fischer Scientific) and 50 μg/ml gentamicin solution (Sigma-Aldrich) and incubated for 1 h at 37°C and 5% CO_2_. The parasites were then transferred to fresh pre-warmed RPMI containing 5% antibiotic-antimycotic solution and 50 μg/ml gentamicin and incubated for 24 h at 37°C and 5% CO_2_.

#### 2.1.2 Collection of Excretory/Secretory Proteins

Medium from the first 24 h containing female or male adults was discarded to prevent contamination with host proteins. Worms were checked daily for viability (motility), immotile worms were removed. Medium was collected and replaced every two to three days. Collected medium was centrifuged at 1000 g for 10 min to pellet possible excreted eggs. The medium supernatant was filtered through a 0.22 µm filter and frozen at -20°C until further concentration. All medium supernatants were concentrated at once using an Amicon filtration unit and a 30kD ultrafiltration membrane (Merck Millipore, US). Medium was exchanged with PBS using the same device. ESP quantity was assessed using the Pierce™ BCA Protein Assay Kit (Thermo Fisher Scientific).

#### 2.1.3 Preparation of ESP

ESP were precipitated using a trichloroacetic acid (TCA) procedure (modified protocol by Thermo Fisher Scientific). Briefly, 71 µg female ESP (duplicates) or 66 µg male ESP (duplicates) were diluted 9 times with dH_2_O, before addition of one fifth (v/v) the volume of sodium deoxycholate 0.15% (w/v), and TCA 72% (w/v). After 10 min of incubation at RT samples were centrifuged 10 min at 5200 *g* at 4°C. Pellets were washed 3 times with ice-cold acetone and centrifuged at 14’000 *g* for 10 min at 4°C. Samples were air dried and resuspended in 100 µl 4% sodium dodecyl sulphate (SDS), Tris-HCl 0.1 M, pH = 7.6, 0.1 M dithiothreitol (DTT) and heated at 95°C for 5 min. Protein content was measured by Qubit protein assay (Thermo Fisher Scientific). Twenty µg protein were processed by filter-aided sample preparation (Wiśniewski et al., 2009). Briefly, samples were mixed with 200 µl 8 M urea/100mM Tris-HCl pH = 8.2, loaded onto Microcon 30 filter units (Millipore) and centrifuged at 14’000 *g* for 30 min at 25°C. Samples were then washed with 200 µl 8 M urea buffer and centrifuged at 14’000 *g* for 30 min at 25°C. One hundred µl 0.05 M iodoacetamide (IAA) was added to the filters, which were then mixed at 600 rpm for 1 min at 25°C on a Thermomixer. Filters were then incubated for 5 min at RT and centrifuged (14’000 *g*) for 15 min at 25°C. Filters were washed 3 times with 8 M urea buffer and twice with 0.5 M NaCl (centrifugation at 14’000 *g*, RT, 12 min). One hundred twenty µl 0.05 M triethylammoniumbicarbonate and 1 µl sequencing grade modified trypsin 0.4 µg/µl (Promega, V5113) were added, mixed for 1 min at 600 rpm and incubated overnight in a wet cell at RT. The next day filters were centrifuged at 14’000 *g* at RT for 15 min. To the flow through 12 µl trifluoroacetic acid (TFA) 5% was added and pH measured using pH test strips. Samples were desalted using C18 stage tips (Rappsilber et al., 2003). Briefly, stage tips were washed and equilibrated with 100% methanol, 60% acetonitrile (ACN)/0.1% TFA and 3% ACN/0.1% TFA. Seventeen µl 3% ACN/0.1% TFA was added to the samples before loading onto the columns and centrifugation at 2000 *g* for 1 min. Samples were washed 2 x with 3% ACN/0.1% TFA and eluted with 60% ACN/0.1% TFA. Samples were then dried to completeness using a speed-vac. Dried samples were resuspended in 3% ACN/0.1% formic acid (FA).

#### 2.1.4 Collection and Preparation of Cuticular Surface Proteins

After 24 h incubation in RPMI medium, parasites were washed 3 times in sterile 37°C PBS. Six replicates of 15 females and 15 males (intact and viable) were transferred into microcentrifuge tubes containing 200 μl sterile PBS. Five μg MS grade trypsin (Pierce™ Trypsin Protease, MS Grade, Thermo Fisher Scientific) was added in each of three replicates from each sex. Three replicates from each sex were incubated without trypsin as controls. Parasites were incubated for 1 h at 37°C. Supernatants were collected and male and female specimens were assessed for viability and integrity. Protein concentration was measured using Qubit protein assay (Thermo Fisher Scientific) and samples digested in solution. Briefly, DTT was added to 5 mM final concentration and samples heated at 60°C for 30 min on a Thermomixer at 600 rpm. IAA was added to 15 mM and samples incubated for 30 min at RT (600 rpm) before quenching with 2 μl of 1 M DTT. Samples were then incubated for 10 min at RT (600 rpm) and pH was adjusted to pH = 8 using 1 M NaOH. Sequencing grade modified trypsin 0.4 µg/µl (Promega, V5113) was added 1:50 (w/w) to each sample and incubated overnight at 37°C (600 rpm). The next day samples were diluted with 600 μl 3% ACN/0.1% TFA for desalting, using Sep-Pak C18 cartridges (Waters). Columns were washed and equilibrated using 100% methanol, 60% ACN/0.1% TFA, and 3% ACN/0.1% TFA before samples were loaded and passed through the column twice. Samples were washed 3 times with 3% ACN/0.1% TFA and eluted with 200 μl 60% ACN/0.1% TFA. Samples were then dried to completeness using a speed-vac and resuspended in 3% ACN/0.1% FA.

#### 2.1.5 LC-MS Analysis

ES and surface peptides were diluted in 3% ACN, 0.1% FA to 1 µg/µl and retention time normalization peptides (iRT, Biognosys) were added (1:20). Data independent analysis (DIA) was performed on a hybrid quadrupole-Orbitrap mass spectrometer (Q Exactive HF, Thermo Fisher Scientific) operated in line with a Acquity UHPLC M-class system (Waters) with a nanoEase M/Z Symmetry C18 trap column (100 A, 5 um, 180 um x 20 mm, Waters) and a nanoEase M/Z HSS C18 T3 analytical column (100 A, 1.8 um, 75 um x 250 mm Column, Waters). Per sample, 2 μl were loaded and elution done by running a linear gradient from 5% to 35% solvent B over 120 min (Solvent A: 0.1% FA in water, solvent B: 0.1% FA in ACN) at 300 nl/min. Peptides were ionized utilizing a nano electrospray ionization (ESI) source (Digital PicoView 565, O/N: DPV-550-565, New Objective, Woburn, MA) and a 10 μm fused-silica spray tip emitter (New Objective, PN). MS1 scans covering 350-1800 m/z were recorded in centroid mode with a resolution of 60’000 using positive polarity and automated gain control (AGC) with a target value of 3e^6^ and a maximum injection time (maxIT) of 200 ms. Every MS1 scan was followed by 35 DIA scans covering a mass range of 400-1100 m/z in 20 m/z isolation windows. DIA scans were recorded in centroid mode with a resolution of 30’000. The AGC target was set to 1e^6^ and the maxIT to 55 ms. Isolated precursors were fragmented with higher-energy collisional dissociation (HCD) at a normalized collision energy (NCE) of 28. Fixed first mass was set to 100 m/z.

Spectronaut (v. 12 and v. 13; Biognosys) was used for label-free protein quantification. More precisely, directDIA analyses based on the UniProt proteomes of *Angiostrongylus costaricensis* and *Angiostrongylus cantonensis* (UP000050601, UP000035642; accessed: 9^th^ of January 2019) were performed because an *A. vasorum* reference genome or proteome was not available. *Angiostrongylus costaricensis* and *A. cantonensis* represented the two most closely related species to *A. vasorum* with available reference proteomes. BGS factory settings were used for analysis. These settings included tryptic specificity, allowing two missed cleavages, carbamidomethyl as a fixed cysteine modification, and oxidation of methionine and protein N-terminal acetylation as variable modifications. Protein groups identified by a single peptide sequence were excluded from analysis. Single hit definition was defined by stripped sequence. FDR was set to 1% for peptide spectrum matches and proteins. The raw mass spectrometry data and Spectronaut outputs have been deposited to the ProteomeXchange Consortium *via* the PRIDE (Vizcaíno et al., 2014) partner repository (dataset identifier PXD027520).

#### 2.1.6 Data Analysis

##### 2.1.6.1 ESP

Mean protein quantities were calculated for female and male ESP separately. Proteins present in one replicate only were excluded from analysis. The obtained protein list was analyzed using Blast2GO (v. 5.2.5) by NCBI blast using blastp (E value < 1e^-3^) to obtain orthologue descriptions for protein identifiers and gene ontology terms associated with them (Conesa et al., 2005). For protease analysis MEROPS (Rawlings et al., 2018) was used. For further analysis specific *C. elegans* orthologues were retrieved *via* NCBI blastp. E-values < 1e^-20^ were considered high quality matches. *C. elegans* orthologues were converted to gene symbols using DAVID (v. 6.8) (Huang et al., 2009) and analyzed with WormEnrichr (Kuleshov et al., 2016) for gene set enrichment analysis. Proteins without *C. elegans* match were analyzed using Pfam (El-Gebali et al., 2019).

##### 2.1.6.2 Surface Proteins

Mean normalization of protein quantities was done for female and male surface samples separately. Proteins present in only one of three replicates were excluded from analysis. To exclude ESP, surface protein quantity was determined by subtracting values of control samples from trypsinized samples. Surface proteins were additionally defined as being present at least 1.2-fold in trypsinized samples compared to control samples.

Proteins were analyzed and orthologues retrieved as described above using Blast2GO (v. 5.2.5). Retrieval of *C. elegans* orthologues and gene set enrichment analysis was done as described for ESP. ESP and surface proteins were qualitatively compared.

#### 2.1.7 Immunolocalization of Cuticular Surface Proteins

To validate proteomic surface data, immunolocalization of two highly abundant surface proteins for which primary antibodies were available was conducted. The following primary antibodies for proteins on female and/or male surface were selected: phosphopyruvate hydratase (syn. enolase 1, ENO) and major sperm protein (MSP). Adult *A. vasorum* specimens were collected and washed as described above. Fourteen male and 15 female nematodes were placed in 10% formalin. An additional 10 females were directly incubated for 4 h with ENO1 recombinant rabbit monoclonal antibody (Thermo Fisher Scientific, MA5-30367, RRID AB_2786110) diluted 1:10 (v/v) in sterile PBS before they were placed in 10% formalin. Nematodes were embedded in paraffin and 5 μm sections were placed on microscope slides, deparaffinised in xylene and rehydrated in an ethanol/distilled water series. Antigen retrieval was performed in 0.1 M sodium citrate (pH = 6.0) and 10 mM Tris, 1 mM EDTA with 0.05% Tween-20 (pH = 9.0) in a steamer for 40 min and 20 min, respectively. Slides were blocked with 10% goat serum diluted in Tris-buffered saline with 0.05% Tween-20 (TBST) for 2 h at RT and washed with TBST 3 times for 5 min. Sections of females were incubated with ENO1 recombinant rabbit monoclonal antibody diluted 1:100 (v/v) in 1% bovine serum albumin (BSA)/TBST and sections of males with Anti-Nematodes Major Sperm Protein monoclonal antibody (Creative Diagnostics, clone 4A5, DMAB9298) diluted 1:50 (v/v) in 1% BSA/TBST. Slide incubated with 1% BSA/TBST only functioned as negative controls. For absorption controls primary antibodies were diluted in 1% BSA/TBST as above and incubated with 1 mg/ml *A. vasorum* somatic antigen for 2 h at 4°C prior to incubation on slides. All slides were incubated overnight at 4°C. After washing, slides were incubated with Alexa Fluor 488 goat anti-mouse IgG antibody (Thermo Fisher Scientific, A-11017) or Alexa Fluor 488 goat anti-rabbit IgG antibody (Thermo Fisher Scientific, A-11070) diluted 1:500 in 1% BSA/TBST and stained with Alexa Flour 568 phalloidin (Thermo Fisher Scientific, A-12380) (1:500) for 2 h at RT in the dark. Slides containing sections of females which were incubated with primary antibodies before fixation were directly incubated with secondary antibodies after blocking. Slides were washed and mounted with Vectashield containing DAPI (Vector Laboratories) and examined with a Leica DMI6000 B inverted fluorescence microscope (Leica Microsystems, Germany) equipped with a Leica DFC365 FX camera (Leica) and recorded using the LAS X software (Leica). Images were compiled in ImageJ (v. 1.53c).

### 2.2 Evaluation of the Effect of ESP on Coagulation in Dog Blood by Rotational Thromboelastometry

**Figure 2:**
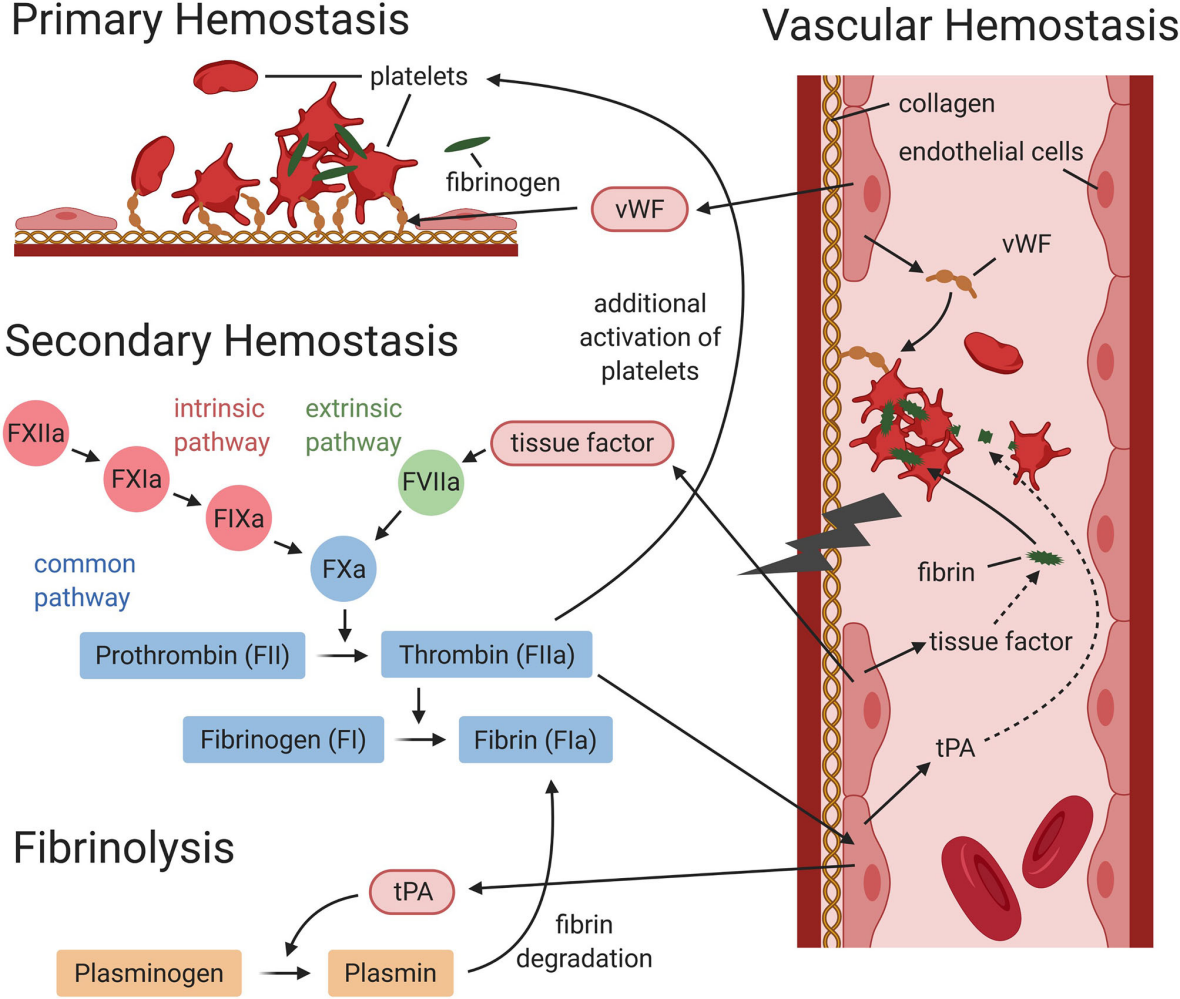
Interaction between vascular, primary and secondary hemostasis, and fibrinolysis (simplified graphic). During primary hemostasis platelets adhere at the vessel injury site, either directly or *via* von Willebrand factor (vWF), to subendothelial collagen. Activated platelets aggregate and fibrinogen forms bridges between activated platelets to form a platelet plug. During secondary hemostasis, the coagulation cascade is activated *via* the intrinsic and/or extrinsic pathway. Both lead to the activation of the common pathway, which will result in the generation of thrombin and fibrin. During fibrinolysis, plasmin induces degradation of fibrin. Vascular endothelial cells express and release factors, which contribute to primary and secondary hemostasis and fibrinolysis. Endothelial cells release von Willebrand factor (needed for platelet adhesion), tissue factor (triggers the activation of the extrinsic pathway), and tissue plasminogen activator (tPA), which mediates plasmin generation. This figure was designed using BioRender.com.

*In vitro* evaluation of ESP on secondary extrinsic hemostasis and fibrinolysis in dog blood was done using rotational thromboelastometry (ROTEM) (**Figure 1B** and **Figure 2**). Blood clotting and fibrinolysis were evaluated over time using ROTEM-Delta (TEM Innovations). Three adult healthy beagle dogs, dog O, dog N, and dog M (2, 4 and 6 years of age) with hematology (full blood count test), coagulation (prothrombin time, partial thromboplastin time, fibrinogen (Clauss method), and ROTEM values within reference ranges were used as blood donors. ROTEM reference values were provided by the Small Animal Clinic, Vetsuisse Faculty, University of Zurich (Jud Schefer et al., 2020).

Whole blood was drawn from the jugular vein into 3.2% sodium citrate (1:9) right before each experiment (animal trial permit no. 299776, 242/17 approved by the Veterinary Office and the Ethics Committee of the Canton of Zurich, Switzerland). Ten μg/ml female ESP or the same volume of PBS as a control was added to collected blood and either incubated for 1 h at 37°C or processed directly.

Right before measuring, recombinant tissue plasminogen activator (tPA, a serine protease, Actilyse^®^, Boehringer Ingelheim) was added in three different concentrations (3.33 μg/ml, 0.33 μg/ml and 0.03 μg/ml) to the blood samples, and one sample containing the same volume of PBS was run as a control. tPA was used to decrease the threshold for lysis onset for evaluation of fibrinolysis, simulating the release of tPA by endothelial cells (since adult *A. vasorum* may lead to mechanical injuries on endothelial cells in the pulmonary artery). After that, 300 μl tPA (or PBS)-treated samples were mixed with 20 μl star-tem (calcium chloride, inhibits citrate and allows coagulation) and 20 μl ex-tem (tissue factor, initiates blood clotting) reagent. Standard operating procedures and the automated pipette program were followed. All ROTEM measurements were done by the same operator. EXTEM measurements were simultaneously run on all four channels and analyzed for 60 min. The following parameters were evaluated for all tracings: clotting time (CT; time in s), clot formation time (CFT; time in s), clot amplitude at 10 and 20 min after CT (A10, A20; in mm), maximum clot firmness (MCF; in mm), and lysis onset time (LOT; in seconds). Data was further analyzed by One-Way ANOVA Tukey’s post test using IBM SPSS Statistics version 27.0.

### 2.3 Evaluation of ESP on Vascular Hemostasis by Cell Stimulation Assay

Canine aortic endothelial cells (CnAOEC, Cell Applications, Inc.) were cultured according to the manufacturer’s instructions with canine endothelial cell growth medium (Cell Applications, Inc.) and kept at 37°C and 5% CO_2_ in a humidified atmosphere. Cell culture experiments were conducted 3 times with cells in passage 2 (**Figure 1C**). For this, cells were seeded into 12-well plates at 25’000 cells/cm^2^ and grown for 2 days to obtain 90% cell confluence. Cells were co-stimulated with 1 or 10 μg/ml freshly collected *A. vasorum* female ESP and 1 ng/ml tumor necrosis factor α (TNFα, Sigma-Aldrich) for 8 and 24 h, since ESP alone did not lead to stimulation. Female ESP were collected, centrifuged and filtered as described above, and immediately concentrated using an Amicon Ultra 15 ml centrifugal filter (10 kDa, Merck Millipore, US). Medium was exchanged to PBS in the same device and the amount of protein was quantified by Qubit protein assay (Thermo Fisher Scientific). ESP endotoxin level was determined prior to cell stimulation experiments using ToxinSensor™ Endotoxin Detection System (GenScript) according to the manufacturer’s instructions and was <0.00001 EU/μg ESP. Non-stimulated cells and cells stimulated with 1 ng/ml TNFα under the same conditions were used as controls. After incubation, endothelial cells were washed with PBS and total RNA was collected using a commercial kit (RNeasy mini kit, Qiagen, Germany) according to the manufacturer’s instructions. RNA quantity was determined with Nanodrop OneC (Thermo Fisher Scientific). Samples were treated with RNase-free DNase I (Thermo Fisher Scientific) before reverse transcription with High Capacity cDNA Reverse Transcription Kit (Applied Biosystems). Quantitative real-time PCR was performed using PowerUp SYBR Green Master Mix (Applied Biosystems) and specific primers for canine glyceraldehyde 3-phosphate dehydrogenase (GAPDH) as the housekeeping gene and internal control (F: 5’ GTCCCCACCCCCAATGTATC 3’, R: 5’ TCCGATGCCTGCTTCACTAC 3’; amplicon: 98 bp), tissue factor (TF) (F: 5’ CATCATCCTGTCTGTGTCTCTG 3’, R: 5’ CTCCAAGGGCACCTTCTTTAT 3’, amplicon: 105 bp), thrombomodulin (TM) [F: 5’ GTGAGCCAGACCGACTATC 3’, R: 5’ GGCACTCTCCGTTTTCGCA 3’, amplicon: 211 bp (Maruyama et al., 2004)], urokinase plasminogen activator (uPA) (F: 5’ CTGCTACGAGGGGAATGGTC 3’, R: 5’ TAGCACCACGGCTTTCTCTG 3’, amplicon: 195 bp), tissue plasminogen activator (tPA) (F: 5’ CACGAGGCGTCTTCTCCTTT 3’, R: 5’ CAGCGGCTAGATGGGTACAG 3’, amplicon: 74 bp), annexin A2 (F: 5’ CTCTCGCAGTGAAGTGGACA 3’, R: 5’ GCTTTCTGGTAGTCGCCCTT 3’, amplicon: 111 bp), and serpin E 1 (syn: plasminogen activator inhibitor-1) (F: 5’ TCATCGTCAACGACTGGGTG 3’, R: 5’ GGCACAGAGACAGTGCTACC 3’, amplicon: 199 bp) at 400 nM of each forward and reverse primers. Each sample (1 ng total RNA) was analyzed in 10 μl reaction mix in triplicates and no-RT controls were run in parallel on Quantstudio 7 Flex Real-Time PCR System (Applied Biosystems). Real time PCR cycle conditions were: 50°C for 2 min, 95°C for 2 min, and 45 cycles at 95°C for 15 s and 60°C for 1 min. Products were validated on a 4% agarose gel. The fold change in cDNA (target gene) relative to the GAPDH endogenous control was determined by the ΔΔCt method (Livak and Schmittgen, 2001). Fold change significance of mRNA expression was determined by One-Way ANOVA Tukey’s post test using IBM SPSS Statistics version 27.0.

## 3 Results

### 3.1 Characterization of *A. vasorum* Excretory/Secretory Proteins and Cuticular Surface Proteins

#### 3.1.1 Excretory/Secretory Proteome

Live adult female and male *A. vasorum* specimens were collected from foxes, taken into culture and ESP collected (**Figure 1A**). Females and males remained viable on average 7 and 9 days, respectively. ESP was collected from 3874 worm days from female worms and 2153 worm days from male worms. A total of 2.1 mg ESP and 0.2 mg ESP was obtained from females and males, respectively.

**Figure 3:**
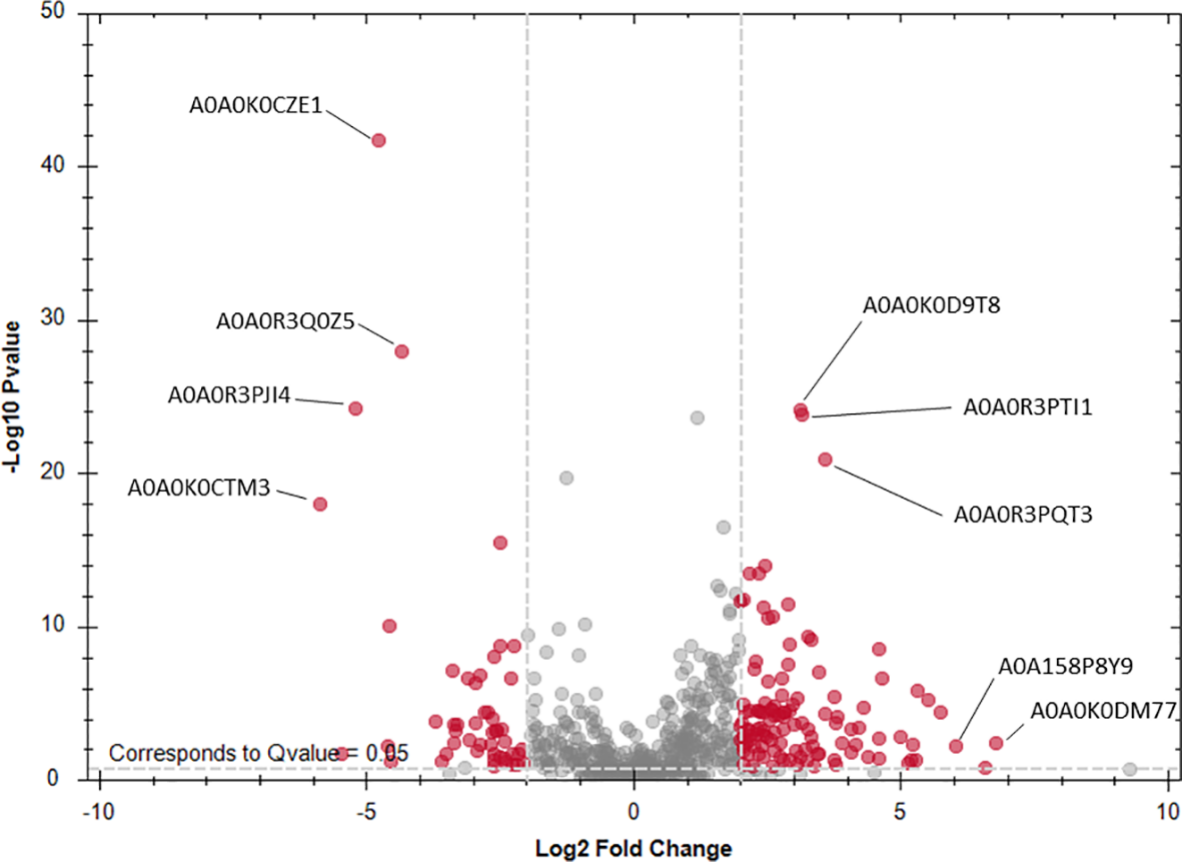
Volcano plot of differentially expressed excretory/secretory proteins of *Angiostrongylus vasorum* obtained by comparing males vs. females. Female ESP: A0A0K0CZE1, A0A0R3Q0Z5, A0A0R3PJI4, A0A0K0CTM3 (orthologue description ‘von Willebrand factor type D domain protein’) Male ESP: A0A0K0D9T8 (orthologue description ‘Kunitz/Bovine pancreatic trypsin inhibitor domain protein’); A0A0R3PTI1 (orthologue description ‘Hsp90 protein’); A0A0R3PQT3 (orthologue description ‘PKS_ER domain-containing protein’); A0A0K0DM77, A0A158P8Y9 (orthologue description ‘MSP domain protein’). Orthologue descriptions obtained from Blast2GO.

Female and male ESP were quantitatively characterized by LC-MS analysis. Referring to the *A. cantonensis* and *A. costaricensis* proteomes, a total of 1069 A*. vasorum* ESP, with 944 proteins in female ESP and 959 proteins in male ESP, were identified. Overall, 834 proteins were shared between females and males, 110 were exclusive to females and 125 exclusive to males. Differentially expressed female and male ESP are displayed in **Figure 3**. Orthologues were retrieved using Blast2GO and were used as description of protein identifiers. Specific *C. elegans* orthologues below the set e-value cut off of 1e^-20^ were retrieved for 731 female ESP and 721 male ESP. Twenty-seven ESP did not have *C. elegans* orthologues, the remainder had e-values above 1e^-20^. Pfam identifiers were available for 7/27 proteins without *C. elegans* match, which were: peptidase_S9_N, fasciclin (2x), tubulin binding cofactor A, G-gamma, glyoxalase, and start domain.

**Figure 4:**
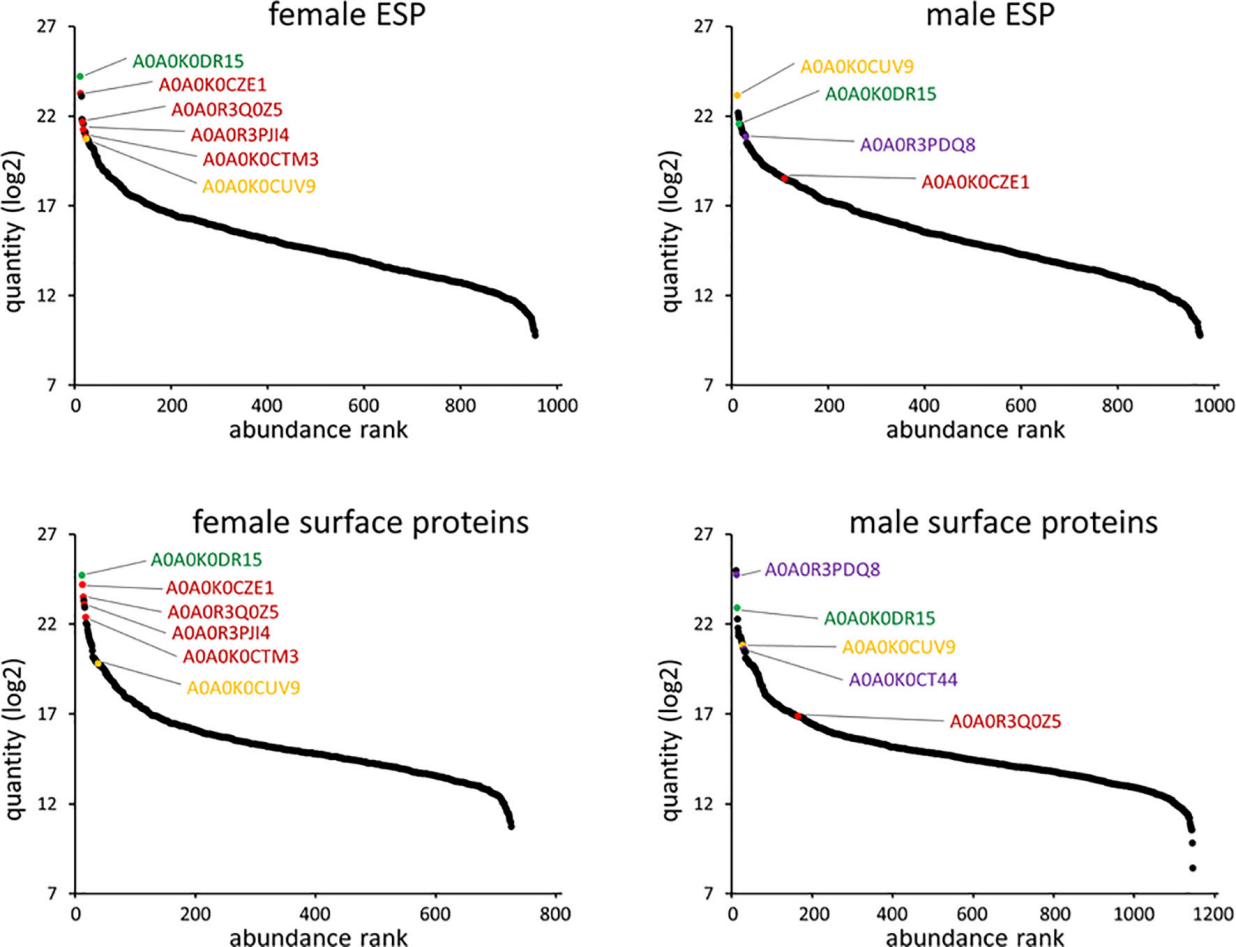
*Angiostrongylus vasorum* female and male protein abundance distribution for ESP and surface proteins. Identifiers that are further discussed or were used for surface immunolocalization are highlighted. A0A0K0CZE1, A0A0R3Q0Z5, A0A0R3PJI4, A0A0K0CTM3 (orthologue description ‘von Willebrand factor type D domain protein’; red); A0A0R3PDQ8, A0A0K0CT44 (orthologue description ‘major sperm protein’; purple); A0A0K0DR15 (orthologue description ‘nematode fatty acid retinoid binding protein’; green); A0A0K0CUV9 (orthologue description ‘enolase’; yellow).

Abundance of female and male ESP and some specific proteins are displayed in **Figure 4**. A0A0K0DR15 (Nematode fatty acid retinoid binding protein) was the most abundant protein in female ESP and the fifth most abundant protein in male ESP. This nematode specific protein is related to parasite development and reproduction and to host infection. The second most abundant protein excreted by females was A0A0K0CZE1 (von Willebrand factor type D domain protein). This protein carries coagulation factor VIII in mammals. The most abundant protein in male ESP was A0A0K0CUV9 (phosphopyruvate hydratase, syn. enolase). Further ESP orthologues from female or male ESP were actin, galectin 2, macrophage migration inhibitory factor, major sperm protein, a-macroglobulin complement component, alpha-2-macroglobulin family protein, HSP90 protein, surfactant protein B, annexin, and galactoside-binding lectin.

Fifty-seven ES products were identified as proteases or proteasome orthologues (5.3% of all proteins) (**Supplementary Material 1**). Several serine (S) type proteases, serine proteinase inhibitors and proteasome protein orthologues were identified. A list of all identified ESP, their abundance and orthologue description are listed in **Supplementary Material 1**.

The top 10 enriched biological processes and KEGG pathways for female and male ESP are displayed in **Table 1**. Most of them are responsible for regular nematode processes (e.g., ‘translation (GO:0006412)’ and ‘glycolysis/gluconeogenesis’). ‘Proteasomal protein catabolic process (GO:0010498)’ was the top 2^nd^ (male) and 4^th^ (female) biological process and ‘proteasome’ the top 4^th^ (female) and 6^th^ (male) pathway. All GO terms related to ESP are listed in **Supplementary Material 1**.

**Table 1:** Top 10 biological processes and KEGG pathways for *Angiostrongylus vasorum* female and male excretory/secretory (ES) and surface proteins (obtained using *C. elegans* orthologue accessions).

Rank	ES proteins								Surface proteins							
	Female				Male				Female				Male			
	Biological process		Pathway		Biological process		Pathway		Biological process		Pathway		Biological process		Pathway	
	Term	Adjusted P-value	Term	Adjusted P-value	Term	Adjusted P-value	Term	Adjusted P-value	Term	Adjusted P-value	Term	Adjusted P-value	Term	Adjusted P-value	Term	Adjusted P-value
**1**	proteasomal ubiquitin-independent protein catabolic process (GO:0010499)	2.00E-08	Ribosome	4.98E-31	proteasomal ubiquitin-independent protein catabolic process (GO:0010499)	7.04E-10	Glycolysis/Gluconeogenesis	1.11E-16	proteasome-mediated ubiquitin-dependent protein catabolic process (GO:0043161)	1.28E-16	Ribosome	7.08E-25	translation (GO:0006412)	2.99E-15	Ribosome	2.02E-25
**2**	ubiquitin-dependent protein catabolic process (GO:0006511)	2.00E-08	Pentose phosphate pathway	3.49E-13	proteasomal protein catabolic process (GO:0010498)	8.02E-10	Ribosome	4.21E-16	ubiquitin-dependent protein catabolic process (GO:0006511)	4.85E-14	Proteasome	8.32E-23	proteasome-mediated ubiquitin-dependent protein catabolic process (GO:0043161)	2.99E-15	Proteasome	7.20E-22
**3**	translation (GO:0006412)	4.52E-08	Glycolysis/Gluconeogenesis	6.09E-13	ubiquitin-dependent protein catabolic process (GO:0006511)	1.11E-09	Pentose phosphate pathway	1.76E-13	embryo development ending in birth or egg hatching (GO:0009792)	3.43E-13	Glycolysis/Gluconeogenesis	7.21E-10	embryo development ending in birth or egg hatching (GO:0009792)	3.87E-14	Citrate cycle (TCA cycle)	3.23E-17
**4**	proteasomal protein catabolic process (GO:0010498)	6.77E-08	Proteasome	9.20E-11	pyruvate metabolic process (GO:0006090)	1.37E-08	Cysteine and methionine metabolism	1.98E-13	proteasomal protein catabolic process (GO:0010498)	3.56E-12	Glutathione metabolism	7.21E-10	ubiquitin-dependent protein catabolic process (GO:0006511)	4.28E-13	Glycolysis/Gluconeogenesis	4.39E-16
**5**	ribosomal large subunit biogenesis (GO:0042273)	8.61E-08	Endocytosis	2.04E-10	proteasome-mediated ubiquitin-dependent protein catabolic process (GO:0043161)	1.37E-08	Phagosome	5.19E-10	translation (GO:0006412)	1.16E-11	Purine metabolism	8.18E-10	proteasomal protein catabolic process (GO:0010498)	2.46E-12	Pyruvate metabolism	7.81E-13
**6**	proteasome-mediated ubiquitin-dependent protein catabolic process (GO:0043161)	8.61E-08	Protein processing in endoplasmic reticulum	2.04E-10	carbohydrate catabolic process (GO:0016052)	1.43E-07	Proteasome	7.15E-10	proteasomal ubiquitin-independent protein catabolic process (GO:0010499)	3.17E-10	Citrate cycle (TCA cycle)	6.47E-09	proteasomal ubiquitin-independent protein catabolic process (GO:0010499)	6.02E-10	Purine metabolism	5.72E-12
**7**	embryo development ending in birth or egg hatching (GO:0009792)	8.61E-08	Phagosome	5.17E-10	nicotinamide nucleotide metabolic process (GO:0046496)	1.43E-07	Pyruvate metabolism	1.08E-09	ERAD pathway (GO:0036503)	4.89E-08	Pentose phosphate pathway	9.32E-09	cytoplasmic translation (GO:0002181)	1.85E-09	Protein processing in endoplasmic reticulum	3.41E-10
**8**	ribosome assembly (GO:0042255)	1.34E-07	Cysteine and methionine metabolism	7.14E-10	proteolysis involved in cellular protein catabolic process (GO:0051603)	1.43E-07	Tyrosine metabolism	1.51E-09	ribonucleoprotein complex assembly (GO:0022618)	1.68E-07	Amino sugar and nucleotide sugar metabolism	1.66E-07	peptide biosynthetic process (GO:0043043)	9.29E-08	Glyoxylate and dicarboxylate metabolism	6.75E-09
**9**	cellular protein metabolic process (GO:0044267)	7.26E-07	Purine metabolism	3.41E-08	embryo development ending in birth or egg hatching (GO:0009792)	1.49E-07	Purine metabolism	2.66E-09	proteolysis involved in cellular protein catabolic process (GO:0051603)	1.68E-07	Phagosome	7.65E-07	protein catabolic process (GO:0030163)	9.29E-08	Pentose phosphate pathway	1.27E-08
**10**	intracellular protein transport (GO:0006886)	1.11E-06	Lysosome	9.72E-08	protein catabolic process (GO:0030163)	1.96E-07	Protein processing in endoplasmic reticulum	2.66E-09	ribosome assembly (GO:0042255)	5.62E-07	Tyrosine metabolism	8.89E-07	proteolysis involved in cellular protein catabolic process (GO:0051603)	1.46E-07	Phagosome	2.10E-08

#### 3.1.2 Cuticular Surface Proteome

All *A. vasorum* specimens remained intact and viable after one h digestion in trypsin. On average 22.7 μg protein was collected from trypsinized female samples and 18.0 μg from female undigested control samples. For males, an average of 11.3 μg were collected from trypsinized samples and 5.8 μg from undigested control samples.

Using a quantitative mass spectrometry approach 705 female surface proteins and 1135 male surface proteins were identified, with 645 proteins common to both females and males, 60 proteins unique to the female surface and 490 unique to the male surface. Orthologues were retrieved and were used as description of protein identifiers. Five hundred thirty-six female surface proteins and 822 male surface proteins had unique *C. elegans* orthologues below the set e-value cut off of 1e^-20^. Twenty-seven proteins had no *C. elegans* orthologue matches. Eight of them identified in Pfam as: peptidase_S9_N, fasciclin, tubulin binding cofactor A (2x), ubiquitin, start domain, GGL domain, and glyoxalase.

Abundance of female and male surface proteins and some specific proteins are displayed in **Figure 4**. Like for ESP, A0A0K0DR15 (Nematode fatty acid retinoid binding protein) was the most abundant protein on female surface. It was also the third most abundant protein on the male surface. Von Willebrand factor type D domain protein orthologues (A0A0K0CZE1 and A0A0R3Q0Z5) were the second and third most abundant on the female surface. The most abundant protein on the male surface was A0A0R3PQU4 (surface-associated antigen 2). A0A0R3PDQ8 (major sperm protein, MSP) was the second most abundant on the male surface, and exclusive to this sex. Further surface protein orthologues were identified as platelet-activating factor acetylhydrolase plasma/intracellular isoform II, galectin 2, macrophage migration inhibitory factor, a-macroglobulin complement component, alpha-2-macroglobulin family protein, HSP90 protein, surfactant protein B, and annexin.

Among surface proteins, 4.7% (56/1195) protease or proteasome orthologues were identified (**Supplementary Material 1**). Fewer S type peptidase orthologues were present on the parasite surface compared to ESP. The orthologue Peptidase, T1 family, was present 6 times and proteasome protein orthologues 10 times. Therefore, 16 out of 56 proteins may be associated with proteasome activity. All surface protein identifiers, their orthologue description and abundance are listed in **Supplementary Material 1**.

**Table 1** contains the top 10 enriched biological processes and KEGG pathways for surface proteins of females and males. The ‘proteasome’ pathway was among the top enriched pathways for both female and male surface proteins. But, as for ESP, regular nematode processes were the most common represented biological processes and pathways. All GO terms associated with surface proteins are available in **Supplementary Material 1**.

#### 3.1.3 Comparison Between ES and Surface Proteins

Overall, 314 proteins were shared between female and male ES and surface proteins. There were 378 proteins of female worms found both in ESP and on the surface. Seventy identified proteins were present exclusively in female ESP and 21 exclusively on the female’s surface. Five hundred twenty-eight proteins of male worms were excreted and located on the surface, 74 were excreted by males only and 287 were exclusive to the male’s surface (**Supplementary Material 2**).

#### 3.1.4 Immunolocalization of Cuticular Surface Proteins

**Figure 5:**
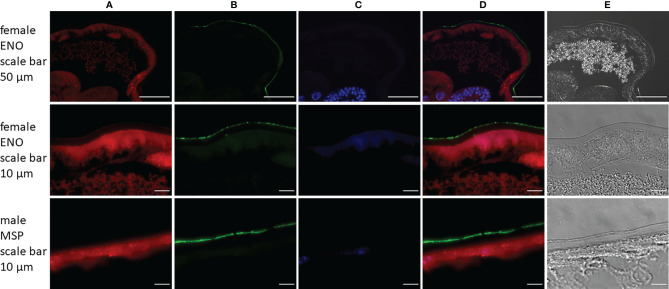
Immunolocalization of enolase (ENO) and major sperm protein (MSP) in sections from *Angiostrongylus vasorum* females or males. Both enolase and major sperm protein [green, panel **(B)**] are localized on the parasites surface. Actin filaments [red, panel **(A)**] were stained using phalloidin as a positive control; DNA [blue, panel **(C)**] was stained with DAPI. Panel **(D)** is a merged image of panels **(A–C)**; panel **(E)** displays transmitted light.

Immunolocalization of two proteins was carried out on sections of *A. vasorum* adult females and males by immunofluorescence to confirm their presence on the outer worm surface. Both enolase (ENO) and major sperm protein (MSP) were abundant proteins on the parasite surface. Enolase ranked 28 in abundance on female surface and MSP ranked 2 on male surface (**Figure 4**). Both proteins were localized in/on the nematode’s cuticular surface coat (green) (**Figure 5**). Negative and absorption controls showed red and blue fluorescent signal for actin and DNA, but no specific enolase or MSP fluorescent signal (**Supplementary Material 3**). This confirms that enolase and MSP are located on the cuticular surface of *A. vasorum* females and males, respectively, and that the direct tryptic surface digestion method led to a reliable surface protein fraction for mass spectrometry.

### 3.2 Evaluation of the Effect of ESP on Coagulation in Dog Blood by Rotational Thromboelastometry

**Figure 6:**
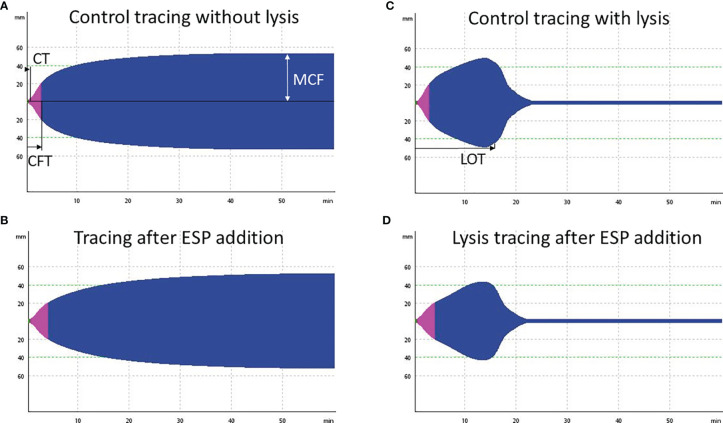
Four exemplary rotational thromboelastometry (ROTEM) EXTEM tracings from dog O. **(A)** Control tracing, incubation for 1 h with PBS and addition of 0.03 μg/ml tissue plasminogen activator (tPA): regular clot formation time (CFT) within 114 seconds. **(B)** Decreased clot firmness (MCF) by 6 mm (11%), and prolonged CFT (187 seconds) after addition of 10 μg/ml *Angiostrongylus vasorum* excretory/secretory proteins (ESP) and incubation for 1 h (including 0.03 μg/ml tPA). **(C)** tPA (0.33 μg/ml) induced fibrinolysis in the same sample as displayed in **(A)**, with a lysis onset time (LOT) of 815 seconds. **(D)** Slightly prolonged LOT of 850 seconds after addition of 10 μg/ml ESP to the same sample as in **(C)**.

Blood samples from three beagle dogs (dogs M, N, O) were treated with 10 μg/ml female ESP and either incubated for 1 h or tested directly to evaluate the effect of ESP on host coagulation using ROTEM (**Figure 1B**). Artificial fibrinolysis using tPA was used to increase the ability to identify differences in fibrinolysis between *A. vasorum* ESP treated and untreated control samples. At 3.33 μg/ml, tPA inhibited coagulation completely (CT: 3594 seconds) (**Supplementary Material 4**). At 0.33 μg/ml tPA, fibrinolysis onset occurred during the 60-min ROTEM run, and at 0.03 μg/ml tPA, blood samples did not show lysis (**Figure 6**).

**Figure 7:**
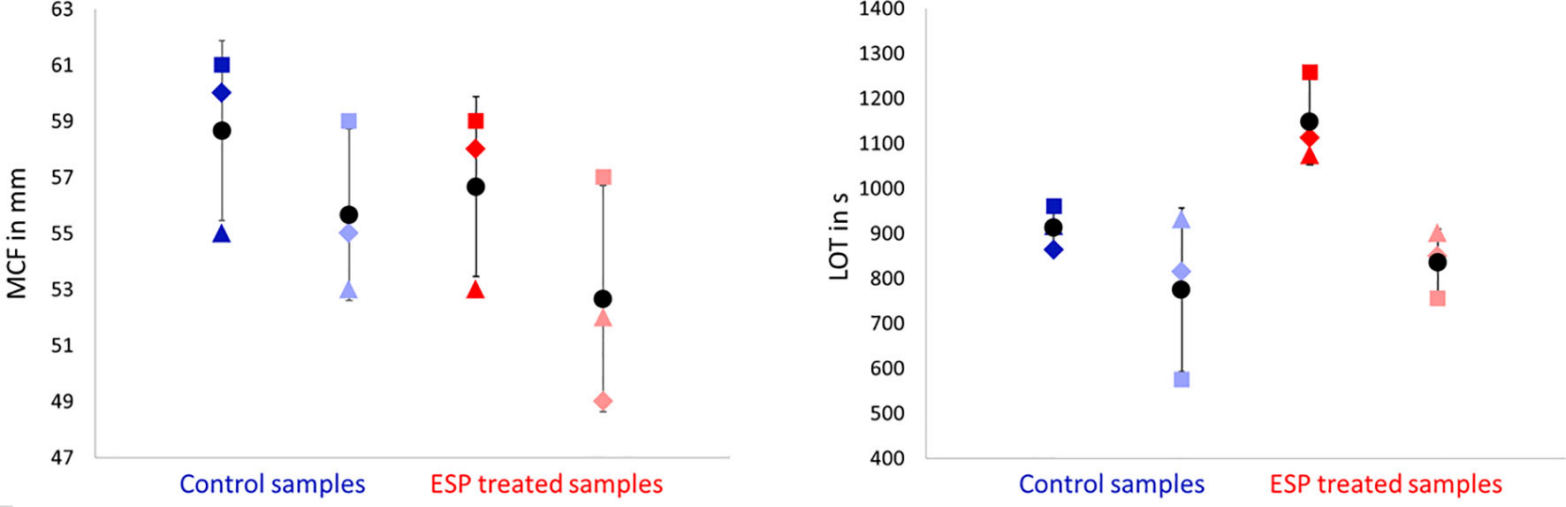
Rotational thromboelastometry (ROTEM) EXTEM MCF (maximum clot firmness, in mm, left side) and LOT (lysis onset time, in s, right side) parameters did not change significantly upon varying sample treatments. ESP treated blood samples (in red) contained 10 μg/ml *Angiostrongylus vasorum* excretory/secretory protein, control blood samples (in blue) contained PBS. Dark blue and dark red symbols indicate no incubation, light blue and light red symbols indicate 1 h incubation (at 37°C). Means (round symbol in black) and standard deviations were calculated based on the measurements from three dogs (dog M: triangle, dog N: square, dog O: diamond).

The addition of *A. vasorum* ESP at 10 μg/ml (with and without incubation) decreased clot firmness (MCF) in all samples but non-significantly (*p* = 0.711 and *p* = 0.887, respectively) (**Figure 7**). There was no common response across individual dogs, but greater alterations were detected in the samples with 1 h incubation before measurement. Changes were most pronounced in blood from dog O after one h incubation (**Figure 7** and **Supplementary Material 4**). The observed findings were even more pronounced when the ESP concentration was increased to 100 μg/ml (done for one dog only): the CFT increased and was above reference values, and MCF decreased further (**Supplementary Material 4**). There was no significant change in fibrinolysis onset time (LOT) after addition of ESP (*p* = 0.904 with incubation, *p* = 0.121 without incubation), even though this was prolonged on average by 148 seconds (**Figure 7**). Only in dog M LOT decreased after addition and incubation with ESP (**Figure 7**). All ROTEM data and parameters are available in **Supplementary Material 4**.

### 3.3 Evaluation of ESP on Vascular Hemostasis by Cell Stimulation Assay

**Figure 8:**
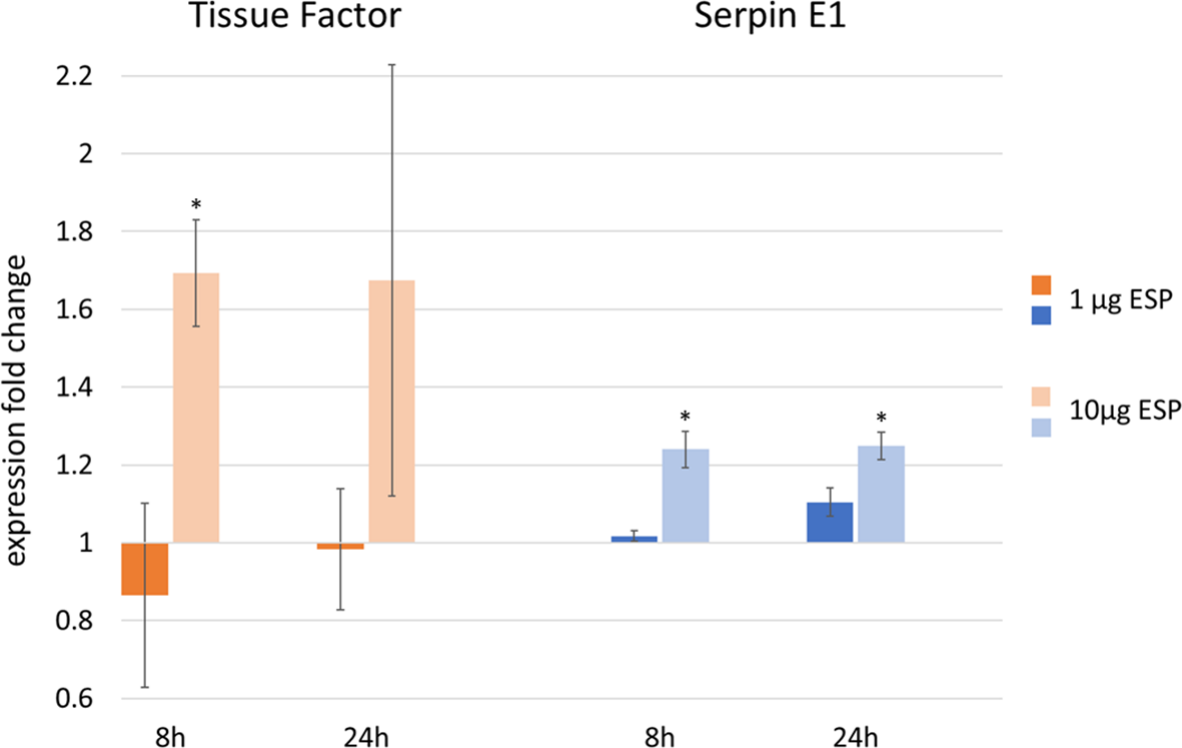
Upregulation of tissue factor (TF) and serpin E1 mRNA expression, as measured by qRT-PCR in canine endothelial cells after stimulation with 1 ng tumor necrosis factor α (TNFα) and 1 μg or 10 μg *Angiostrongylus vasorum* excretory/secretory proteins (ESP) incubated for 8 or 24 h (data based on 3 replicates). Cells stimulated with 1 ng TNFα only represent the control values of 1. *p < 0.05, One-Way ANOVA Tukey’s post test.

CnAOEC were stimulated with TNFα and 1 μg or 10 μg *A. vasorum* female ESP. Transcript expression of GAPDH, TF, TM, uPA, tPA, annexin A2 and serpin E1 was analyzed by qRT-PCR, fold changes determined by the ΔΔCt method and analyzed by One-Way ANOVA. Experiments were conducted thrice in separate runs. After 8 and 24 h of incubation TNFα alone led to a maximum fold change of 3.40 in TF, 0.68 in TM, 1.43 in uPA, 1.12 in serpin E1, 1.08 in annexin A2, and 0.91 in tPA (**Supplementary Material 5**). One μg ESP did not lead to any significant fold changes and was not above 1.11 or below 0.86 for any of the 6 targets at either time point (**Supplementary Material 5**). Ten μg ESP led to a significant TF mRNA expression change of 1.69 at 8 h (*p* = 0.039) but not at 24 h (fold change: 1.67, *p* = 0.244) (**Figure 8**). Serpin E1 expression changed significantly and was 1.24 at 8 h (*p* < 0.001) and 1.25 at 24 h (*p* = 0.003) after stimulation with 10 μg ESP (**Figure 8**). uPA, tPA, TM and annexin A2 mRNA expression did not significantly increase or decrease after stimulation of cells with 10 μg ESP: after 8 h incubation, mRNA expression changes were 0.92, 0.98, 0.98 and 1.07 for uPA, tPA, TM and annexin A2, respectively, and after 24 h, they were 0.86, 0.96, 0.92 and 1.05, respectively (**Supplementary Material 5**). Gel pictures of final products are available in **Supplementary Material 6**.

## 4 Discussion

This study presents the results of the first proteomic analysis of *A. vasorum* ESP and cuticular surface proteins and discusses putative interactors with host coagulation or host immune response. Previous studies dealt with crude proteins from *A. vasorum* adults and larvae, with the goal to identify specific antigens for immunodiagnosis (Oliveira Vasconcelos et al., 2007; Jefferies et al., 2011). Jefferies et al. (2011) detected 11 immuno-reactive proteins, including fatty acid and retinol-binding proteins, major sperm protein and actin, proteins which were also identified in *A. vasorum* ESP and on their surface. In this study, a large number of ESP and surface proteins from adult specimens were characterized, with a large fraction being present in both *A. vasorum* ESP and on the surface. The fraction of surface proteins analyzed here represents the surface coat, which is loosely apposed to the epicuticule. This labile layer is released by the excretory system and from gland cells. The surface coat therefore represents an active, dynamic and responsive structure (Blaxter et al., 1992). Surface proteins are continuously shed into the environment and play a key role in the interaction between the nematode and host. They may act as a removable coat to protect the nematode from immune cells or antibodies, and to deflect from the nematode (Blaxter et al., 1992). Therefore, qualitatively, surface proteins overlap substantially with ESP (Davies and Curtis, 2011), as observed in this study. Moreover, the continuous shedding of the surface coat makes it a difficult structure to preserve for e.g., microscopy: this coat can be lost upon preparation (Blaxter et al., 1992), which explains the non-continuous signal obtained on the parasite surface by fluorescence microscopy.

Enolase and major sperm protein were described in ESP of other nematodes (Hewitson et al., 2008) and were also identified on the tegument of e.g., *Schistosoma bovis* (a trematode) (Pérez-Sánchez et al., 2008). The images obtained by immunofluorescence microscopy indicate the presence of enolase and major sperm protein on the surface of female and male nematodes, respectively. This therefore confirms that direct tryptic surface digestion of live worms results in reliable collection of surface proteins.

Von Willebrand factor type D domain protein orthologues (A0A0K0CZE1, A0A0R3Q0Z5, A0A0R3PJI4, A0A0K0CTM3) represent a highly abundant protein group in *A. vasorum* female ESP and on its surface. The mammalian von Willebrand factor protein is a large glycoprotein and plays a central role in primary hemostasis and thrombosis (**Figure 2**). D domains are conserved and represent the binding site for coagulation factor VIII, which is transported to platelet plugs. Four D domains are present in mammalian von Willebrand factor (Springer, 2014). Considering the abundant release of von Willebrand factor type D domain protein orthologues from female *A. vasorum* and the frequent clinical sign of bleeding observed in *A. vasorum* infected dogs, this parasite protein class may play a significant role in the pathogenesis of *A. vasorum* induced coagulopathies. So far, among helminths, von Willebrand domains have only been described in the *Fasciola hepatica* tegument proteome (Wilson et al., 2011).

The von Willebrand factor type D domain protein is furthermore present in vitellogenin, an egg yolk precursor protein, which is relevant for all oviparous species, like some nematodes. It is possible that the identified von Willebrand factor type D domain protein orthologues are part of an *A. vasorum* vitellogenin-like protein. In *C. elegans* six vitellogenin genes are present (Blumenthal et al., 1984) and in *Heligmosomoides polygyrus*, an intestinal nematode of rodents, vitellogenin represents an abundant adult ESP (Hewitson et al., 2011). Hewitson et al. (2011) suggested that this protein is present in uterine fluid or that it diffuses from eggs, also in culture. This protein group, however, was also present in *A. vasorum* male ESP and on the male surface, indicating that this protein is not specifically associated with female metabolism.

A large number of *A. vasorum* ESP and surface proteins were identified as proteases, protease inhibitors, or proteasomes, including T1 family proteases and proteasome orthologues. Proteases are described to help helminths evade from the immune response but may also induce inflammatory responses (McKerrow et al., 2006) and are common in helminth ESP and surfaces (Yatsuda et al., 2003; Hewitson et al., 2011; Wilson et al., 2011; Cui et al., 2013). Also the sister species *A. cantonensis and A. costaricensis* have 14 T1 family peptidase homologues (data obtained from MEROPS database). In this study, the proteasomal catabolic processes were among the most upregulated biological processes in *A. vasorum* ESP and surface proteins, and ‘proteasome’ was among the top enriched pathways in surface proteins. Parasite serine type proteases are involved in host invasion, meanwhile host serine proteases are relevant for digestion, coagulation and fibrinolysis (Dzik, 2006). In particular, the mammalian coagulation cascade is composed of several serine proteases. For instance, the S1 protease family contains several coagulation factors and complement components (MEROPS database). Interestingly, serine type proteases such as S1 and S28 protease orthologues were also produced by *A. vasorum*. Serine proteases released by *A. vasorum* may therefore interfere with host coagulation at several key points and may for instance activate plasma prekallikrein *via* S28 proteases (Shariat-Madar et al., 2004), or mimic coagulation factors.

Additionally to helminth proteases, helminth derived protease inhibitors were shown to protect against host derived proteases and are believed to downregulate host immunity and inflammatory responses, and may also act as anticoagulants (McKerrow et al., 2006). *Angiostrongylus vasorum* releases several protease inhibitor orthologues and expresses them on the surface. In analogy, blood-feeding hookworms have several serine specific inhibitors, which were shown to interfere with the blood coagulation cascade and facilitate blood feeding (Cappello et al., 1995). These serine inhibitors were localized in different organs of the nematodes and inhibit coagulation factor X, the connection point for the extrinsic and intrinsic coagulation pathways (**Figure 2**) (Cappello et al., 1995; Mieszczanek et al., 2004).

Lectins and galectins are involved in various immune processes. Nematode lectins share sequences with mammalian immune cell lectins (Loukas and Maizels, 2000) and may bind nematode carbohydrates to ‘hide’ from host immune cells (Hewitson et al., 2009). Galectins are very common components of nematode ESP (Bennuru et al., 2009; Hewitson et al., 2009; Hewitson et al., 2011; González-Miguel et al., 2012), and the most abundant ESP in *Brugia malayi* (Hewitson et al., 2008; Bennuru et al., 2009). Galectins can accumulate at the endothelial interface and promote endothelial cell proliferation (Sanford and Harris‐Hooker, 1990). In particular, galectin 1 and 3, which however were not present in *A. vasorum* ESP, are known to bind to von Willebrand factor and to coagulation factor VIII (O’Sullivan et al., 2016). This nevertheless may suggest that *A. vasorum* galectins may modulate host coagulation, and the galactoside-binding lectin orthologues abundantly released by *A. vasorum* could therefore also represent proteins that interact with host coagulation.

Fibrinolysis (the breakdown of blood clots) is a highly regulated process and an essentially interconnected part of the coagulation cascade (Cesarman-Maus and Hajjar, 2005). The onset of fibrinolysis occurs trough tPA converting plasminogen to active plasmin (**Figure 2**). Host plasminogen, however, is also utilized by infectious agents during invasion (Boyle and Lottenberg, 1997) and several helminths interact with the host fibrinolytic system by expressing or secreting plasminogen receptors (González-Miguel et al., 2016). In fact, specific helminth proteins have been identified as plasminogen binding proteins, some of which may also generate plasmin (Ramajo-Hernández et al., 2007; Figuera et al., 2013; González-Miguel et al., 2013). Plasmin is relevant within the coagulation cascade for the lysis of fibrin clots. Such plasminogen binding proteins are therefore abundant among helminths and present in ESP or the surface of several nematodes and trematodes (Hewitson et al., 2008; Pérez-Sánchez et al., 2008; Hernández-González et al., 2010). Enolase (identified in *A. vasorum* ESP and on its surface), for example, is a highly conserved protein and a multifunctional glycolytic enzyme, which may also act as a plasminogen receptor on a variety of cells (Pancholi, 2001). Several further helminth plasminogen binding proteins were present in *A. vasorum* ESP and/or surface proteins, such as actin, GAPDH and fructose-bisphosphate aldolase. In blood dwelling parasites, plasminogen binding capacity (and generation of plasmin) may represent a survival mechanism to avoid clot formation around the pathogen (Ramajo-Hernández et al., 2007; González-Miguel et al., 2015c). However, increased generation of plasmin over a long period of time may lead to coagulopathies including hyperfibrinolysis (Kolev and Longstaff, 2016), which was also identified in bleeding *A. vasorum* infected dogs (Sigrist et al., 2017). This is where proteins such as alpha 2-macroglobulin, expressed by the host but also released by *A. vasorum* and expressed on its surface, potentially intervene. Alpha 2-macroglobulin can regulate fibrinolysis by irreversibly binding to the active site of plasmin (Cesarman-Maus and Hajjar, 2005). Eventually, the activity of several helminth-derived interactors with host coagulation or fibrinolysis has been assessed (Ramajo-Hernández et al., 2007; González-Miguel et al., 2015b; Diosdado et al., 2020). Orthologues of some of these potential activators were identified in *A. vasorum* ESP or on its surface. Whether these specific proteins and proteases are present in an active form requires further investigation. This study evaluated the activity of ESP on host coagulation *in vitro* by viscoelastic methods and canine endothelial cell stimulation assays.

Viscoelastic methods such as thromboelastometry or thromboelastography (TEG) are regularly used as diagnostic and research tools. They allow studying clotting and fibrinolysis in whole blood (Longstaff, 2018). This study showed that ROTEM can be used for the evaluation of *A. vasorum* ESP on clotting and fibrinolysis in dog whole blood: by adding tPA to ESP spiked blood samples the release of tPA by endothelial cells was mimicked. Artificial fibrinolysis was observed, as previously reported with dog samples (Fletcher et al., 2016), demonstrating that *A. vasorum* ESP has no significant effect on host fibrinolysis *in vitro*. However, the ROTEM analysis was limited to 3 healthy dogs (with normal coagulation parameters) of the same breed. Response to the addition of ESP was individual, without a common trend. This suggests that the response upon natural infection may also depend on the individual animal and its coagulation response in addition to, likely, the infective dose and chronicity of the disease. Accordingly, the findings obtained by ROTEM with *A. vasorum* ESP spiked blood samples do not fully reflect the situation in naturally infected dogs. So far, two studies focusing on coagulation in *A. vasorum* naturally infected dogs included viscoelastic methods. Interestingly, in both hypocoagulation and hyperfibrinolysis were the main findings (Adamantos et al., 2015; Sigrist et al., 2017), often diagnosed in combination with clinical signs of bleeding (Sigrist et al., 2018; Sigrist et al., 2021). Hypocoagulation after addition of ESP to dog blood *in vitro* was not observed. However, parameters such as slightly prolonged CFT and marginally decreased MCF could suggest that the induction of hypocoagulation through ESP is possible. In natural infections hypofibrinogenemia was observed (Adamantos et al., 2015; Sigrist et al., 2017) as a result of increased fibrinogen consumption, associated with continuous clot lysis (Sigrist et al., 2017). *In vitro*, the addition of *A. vasorum* ESP did not decrease lysis onset time, but rather prolonged it slightly in two out of three dogs.

Stimulation of canine endothelial cells with *A. vasorum* ESP only showed minor mRNA expression changes of factors relevant for coagulation and fibrinolysis. TF and serpin E1 were significantly increased upon stimulation with a high concentration of ESP. Increased expression of TF induces increased secondary hemostasis through the extrinsic activation pathway together with coagulation factor VII, causing increased thrombin formation through activation of coagulation factor X (**Figure 2**) (McVey, 2016). Serpin E1, instead, is a plasminogen activator inhibitor and inhibits the activation of fibrinolysis (Rau et al., 2007). tPA and uPA are the main activators of fibrinolysis, and annexin A2 is a cofactor for plasmin generation involved in vascular fibrinolysis (Cesarman-Maus and Hajjar, 2005; Flood and Hajjar, 2011), however none of these factors were increased, further indicating that ESP does not directly induce fibrinolysis. Also the TM expression was not significantly altered. TM is an anticoagulant, inhibiting thrombin from interacting with fibrinogen (Weiler and Isermann, 2003). An increased expression of TF and simultaneous decrease of TM often go hand in hand in endothelial cells upon stimulation with inflammatory proteins or pathogens (Key et al., 1990; Schmidt et al., 2006), as observed by TNFα stimulation as a positive control and co-stimulant. Additional stimulation with ESP, however, did not amplify this effect on TM. These findings suggest that the contribution of ESP on vascular hemostasis upon infection is minor. However, it cannot be ruled out that prolonged exposure, such as in chronic infections, may eventually lead to more pronounced expression changes and release of procoagulant components (e.g., TF) or profibrinolytic factors (e.g., tPA, uPA or annexin A2).

Interestingly, *Dirofilaria immitis*, another nematode parasitizing the heart and pulmonary arteries of dogs, is not known to cause bleeding disorders in dogs but its ESP enhanced tPA and uPA protein expression in stimulated endothelial cells and decreased serpin E1 protein expression (González-Miguel et al., 2012; González-Miguel et al., 2015a), which is in contrast to our findings. Specific *D. immitis* proteins such as actin and fructose-bisphosphate aldolase were able to increase tPA and uPA protein expression in endothelial cells (González-Miguel et al., 2015c), and surface associated *D. immitis* GAPDH and galectin also increased the uPA protein expression in endothelial cells (González-Miguel et al., 2015b). *Dirofilaria immitis* ESP or surface proteins were in addition capable of binding and activating plasminogen (González-Miguel et al., 2012; González-Miguel et al., 2013) and *D. immitis* ESP caused endothelial cell proliferation and migration *in vitro* (González-Miguel et al., 2015a). The *D. immitis* studies, however, were based on expression of proteins, and not on mRNA expression as in this study: a direct comparison is therefore difficult. *Dirofilaria immitis* is also a much larger parasite than *A. vasorum* and may consequently need to interact with the host in a more pronounced manner. Additional research will be needed to study the differences upon endothelial cell stimulation of these two cardiopulmonary nematodes of canids.

The fact that addition of *A. vasorum* ESP did not increase fibrinolysis in dog blood nor increase expression of fibrinolysis factors in endothelial cells suggests that hyperfibrinolysis observed in *A. vasorum* infected dogs is not a result of direct fibrinolytic activity by *A. vasorum* products. The increase of a fibrinolysis inhibitor after ESP stimulation in endothelial cells rather suggests inhibition of fibrinolysis. The minor expression changes in components contributing to vascular hemostasis indicates minor influence of ESP on host vascular hemostasis. Further studies on specific *A. vasorum* ESP or surface proteins that have been identified are needed to evaluate their targeted effect on host coagulation and fibrinolysis. Therefore, *A. vasorum* adult ESP alone do not induce bleeding disorders in infected dogs. Nevertheless, it cannot be ruled out that *A. vasorum* adult ESP contribute to coagulopathies and fibrinolysis. Both ESP and surface proteins may induce coagulopathies or fibrinolysis in combination with other processes *in vivo* such as the direct contact of the parasite and its surface proteins with the endothelium, through mechanical stress, or together with proteins released by decaying parasites. Additionally, developmental stages such as eggs, first or third stage larvae may contribute to fibrinolysis and coagulation. Egg shells persisting in the host likely induce immune and therefore inflammatory reactions, and migrating larvae cause tissue damage leading to local inflammation (Schnyder et al., 2010). In massive and/or chronic infections, this possibly has a systemic effect. All these factors may have an impact on the onset of bleeding disorders observed in *A. vasorum* infected dogs.

## 5 Conclusion

A complex mixture of proteins, proteases and their inhibitors are released by *A. vasorum*. Several ESP and surface proteins may interfere with the host and its coagulation and fibrinolysis. The analysis of ESP in dog blood did not show an impact on fibrinolysis onset, and changes in canine endothelial cells exposed to ESP suggest only minor interaction with host vascular hemostasis. ESP may stimulate extrinsic activation of secondary hemostasis by increased expression of TF but do not lead to increased profibrinolytic components, and rather to an increase of fibrinolysis inhibitors. *Angiostrongylus vasorum* ESP therefore are suggested to contribute to coagulopathies observed upon infection in dogs, likely in combination with other factors such as mechanical stress caused by adults, or damage caused through migration of other parasitic life stages. Bleeding disorders observed in *A. vasorum* infected dogs likely result from a multifactorial response of the host to this parasitic infection.

## Data Availability Statement

The datasets presented in this study can be found in online repositories. The names of the repository/repositories and accession number(s) can be found in the article.

## Ethics Statement

The animal study was reviewed and approved by the Veterinary Office and the Ethics Committee of the Canton of Zurich, Switzerland under permit number no. 299776, 242/17.

## Author Contributions

NG, LT, TK, and MS conceived the study. NG carried out the experiments and analyzed microscopy and cell stimulation data. NG, LT and TK analyzed proteomics data. NG and LA analyzed ROTEM data. NG, LT, TK, LA, and MS contributed to experimental design and the interpretation of results. LT and MS supervised the project. TK and LA were advisors of the project. NG wrote the manuscript with support from LT, MS, and TK. All authors contributed to the article and approved the submitted version.

## Funding

Funding was provided by the ‘Forschungskredit’ FK-20-060 and FK-16-059 from the University of Zurich, Switzerland in the frame of financial support to NG. and LT. Bayer Vital GmbH, Business Unit Animal Health, Germany, supported NG during her PhD studies. The funders had no role in study design, data collection and analysis, decision to publish, or preparation of the manuscript.

## Conflict of Interest

The authors declare that the research was conducted in the absence of any commercial or financial relationships that could be construed as a potential conflict of interest.

## Publisher’s Note

All claims expressed in this article are solely those of the authors and do not necessarily represent those of their affiliated organizations, or those of the publisher, the editors and the reviewers. Any product that may be evaluated in this article, or claim that may be made by its manufacturer, is not guaranteed or endorsed by the publisher.

## Abbreviations

ESP, excretory/secretory proteins; ES, excretory/secretory; LC-MS, liquid chromatography - mass spectrometry; DIC, disseminated intravascular coagulation; ENO, enolase; MSP: major sperm protein; ROTEM, rotational thromboelastometry; PBS, phosphate-buffered saline; TCA, trichloroacetic acid; DTT, dithiothreitol; SDS, sodium dodecyl sulphate; IAA, iodoacetamide; CAN, acetonitrile; TFA, trifluoroacetic acid; FA, formic acid; DIA, data independent analysis; ESI, electrospray ionization; AGC, automated gain control; maxIT, maximum injection time; HCD, higher-energy collisional dissociation; NCE, normalized collision energy; TBST, tris-buffered saline with 0.05% Tween-20; BSA, bovine serum albumin; CT, clotting time; CFT, clot formation time; MCF, maximum clot firmness; LOT, lysis onset time; CnAOEC, canine aortic endothelial cells; TNFα, tumor necrosis factor α; GAPDH, glyceraldehyde 3-phosphate dehydrogenase; TF, tissue factor; TM, thrombomodulin; uPA, urokinase plasminogen activator; tPA, tissue plasminogen activator; vWF, von Willebrand factor; GO, gene ontology; TEG, thromboelastography.
