# Supplementary material for: The Angiostrongylus vasorum Excretory/Secretory and Surface Proteome Contains Putative Modulators of the Host Coagulation
Source: Front Cell Infect Microbiol. 2021 Nov 2;11:753320. doi: 10.3389/fcimb.2021.753320 (PMC8593241; doi:10.3389/fcimb.2021.753320)

Supplementary Material

## Supplementary Figures

**Supplementary Material 2.** Comparison between female and male *Angiostrongylus vasorum* excretory/secretory proteins (ESP) and surface proteins

**
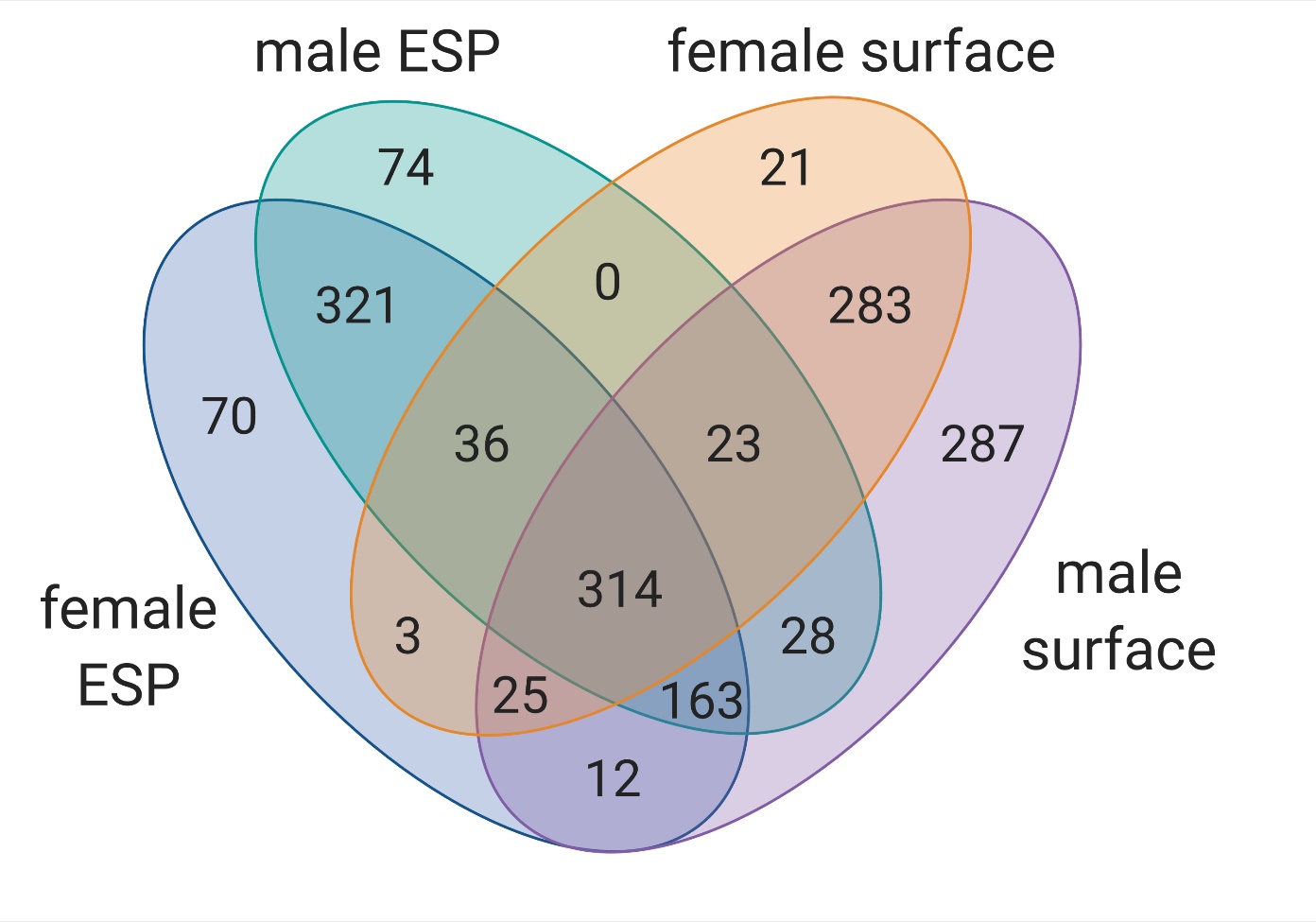
**

**Supplementary Material 3.** Immunolocalization negative and absorption controls of enolase (ENO) and major sperm protein (MSP) on *Angiostrongylus vasorum* female and male adult cuticular surfaces showing red and blue fluorescent signal for actin and DNA, but no specific enolase or MSP fluorescent signal.

**
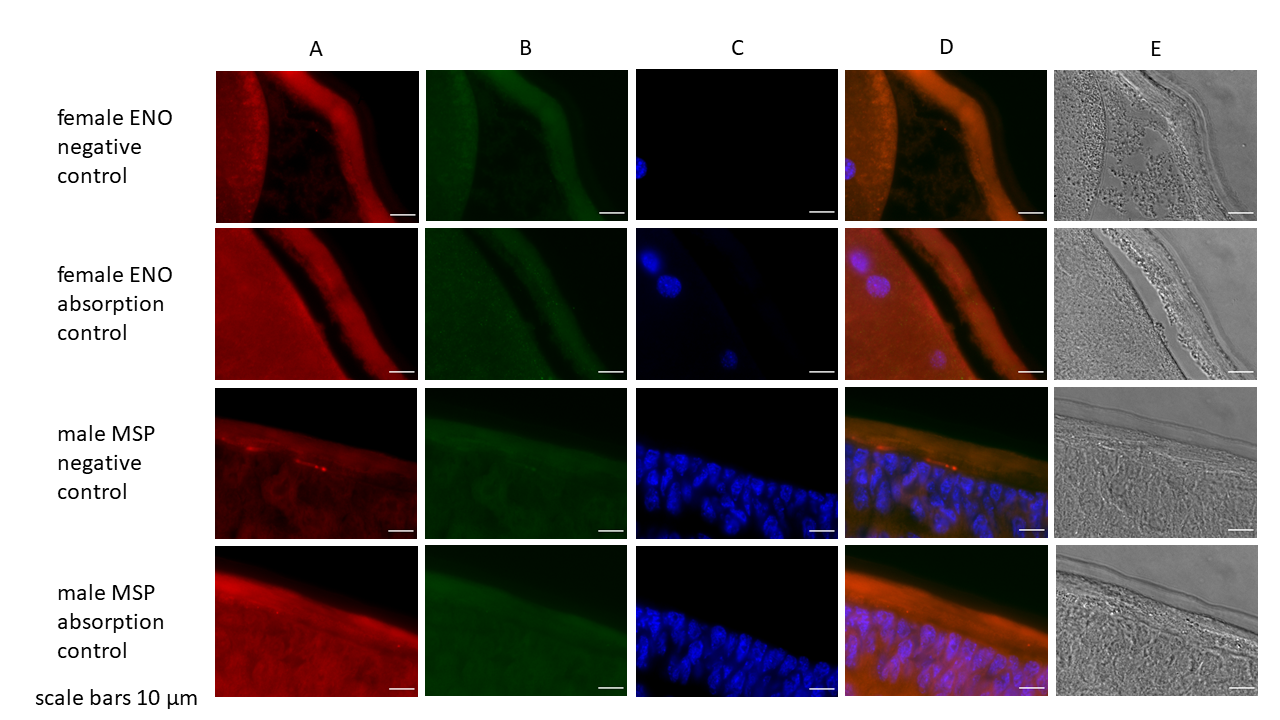
**

**Supplementary Material 6.** Gel electrophoresis (4% agarose gel) of qPCR products from canine endothelial cells stimulated with *Angiostrongylus vasorum* excretory/secretory proteins


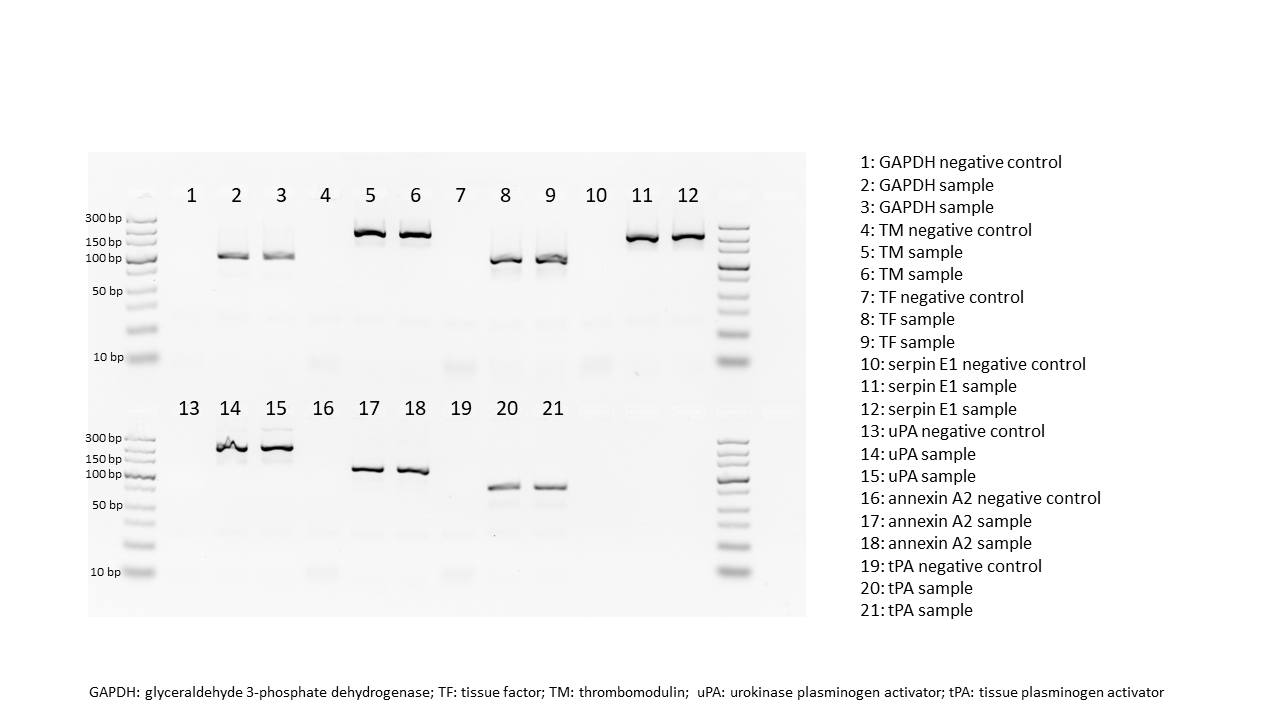

Supplement: Supplementary file 1 [file DataSheet_1.zip › Supplementary Material 2, 3, 6.docx]
